# Association of blood group O with a recurrent risk for acute lower gastrointestinal bleeding from a multicenter cohort study

**DOI:** 10.1038/s41598-024-64476-9

**Published:** 2024-06-17

**Authors:** Sho Suzuki, Naoyuki Tominaga, Tomonori Aoki, Eiji Sadashima, Tadashi Miike, Hiroshi Kawakami, Katsumasa Kobayashi, Atsushi Yamauchi, Atsuo Yamada, Jun Omori, Takashi Ikeya, Taiki Aoyama, Yoshinori Sato, Takaaki Kishino, Naoki Ishii, Tsunaki Sawada, Masaki Murata, Akinari Takao, Kazuhiro Mizukami, Ken Kinjo, Shunji Fujimori, Takahiro Uotani, Minoru Fujita, Hiroki Sato, Toshiaki Narasaka, Junnosuke Hayasaka, Tomohiro Funabiki, Yuzuru Kinjo, Akira Mizuki, Shu Kiyotoki, Tatsuya Mikami, Ryosuke Gushima, Hiroyuki Fujii, Yuta Fuyuno, Takuto Hikichi, Yosuke Toya, Kazuyuki Narimatsu, Noriaki Manabe, Koji Nagaike, Tetsu Kinjo, Yorinobu Sumida, Sadahiro Funakoshi, Kiyonori Kobayashi, Tamotsu Matsuhashi, Yuga Komaki, Mitsuru Kaise, Naoyoshi Nagata

**Affiliations:** 1 Department of Gastroenterology and Hepatology, Center for Digestive Disease and Division of Endoscopy, University of Miyazaki Hospital, Miyazaki, Japan; 2 Department of Gastroenterology, Saga-Ken Medical Centre Koseikan, Saga, Japan; 3 Department of Gastroenterology, Graduate School of Medicine, The University of Tokyo, Tokyo, Japan; 4 Department of Medical Research Institute, Saga-Ken Medical Centre Koseikan, Saga, Japan; 5 Department of Gastroenterology, Tokyo Metropolitan Bokutoh Hospital, Tokyo, Japan; 6 Department of Gastroenterology and Hepatology, Kitano Hospital, Tazuke Kofukai Medical Research Institute, Osaka, Japan; 7 Department of Gastroenterology, Nippon Medical School, Graduate School of Medicine, Tokyo, Japan; 8 Department of Gastroenterology, St Luke’s International University, Tokyo, Japan; 9 Department of Gastroenterology, Hiroshima City Asa Citizens Hospital, Hiroshima, Japan; 10 Division of Gastroenterology, Department of Internal Medicine, St Marianna University School of Medicine, Kanagawa, Japan; 11 Department of Gastroenterology and Hepatology, Center for Digestive and Liver Diseases, Nara City Hospital, Nara, Japan; 12 Department of Gastroenterology, Tokyo Shinagawa Hospital, Tokyo, Japan; 13 Department of Endoscopy, Nagoya University Hospital, Aichi, Japan; 14 Department of Gastroenterology, National Hospital Organization Kyoto Medical Center, Kyoto, Japan; 15 Department of Gastroenterology, Tokyo Metropolitan Cancer and Infectious Diseases Center Komagome Hospital, Tokyo, Japan; 16 Department of Gastroenterology, Oita University, Oita, Japan; 17 Department of Gastroenterology, Fukuoka University Chikushi Hospital, Fukuoka, Japan; 18 Department of Gastroenterology, Chiba Hokusoh Hospital, Nippon Medical School, Chiba, Japan; 19 Department of Gastroenterology, Japanese Red Cross Shizuoka Hospital, Shizuoka, Japan; 20 Division of Endoscopy and Ultrasonography, Department of Clinical Pathology and Laboratory Medicine, Kawasaki Medical School General Medical Center, Okayama, Japan; 21 Division of Gastroenterology, Graduate School of Medical and Dental Sciences, Niigata University, Niigata, Japan; 22 Department of Gastroenterology, University of Tsukuba, Ibaraki, Japan; 23 Division of Endoscopic Center, University of Tsukuba Hospital, Ibaraki, Japan; 24 Department of Gastroenterology, Toranomon Hospital, Tokyo, Japan; 25 Department of Emergency Medicine, Fujita Health University Hospital, Aichi, Japan; 26 Emergency and Critical Care Center, Saiseikai Yokohama Tobu Hospital, Kanagawa, Japan; 27 Department of Gastroenterology, Naha City Hospital, Okinawa, Japan; 28 Department of Internal Medicine, Tokyo Saiseikai Central Hospital, Tokyo, Japan; 29 Department of Gastroenterology, Shuto General Hospital, Yamaguchi, Japan; 30 Division of Endoscopy, Hirosaki University Hospital, Hirosaki, Aomori Japan; 31 Department of Gastroenterology and Hepatology, Graduate School of Medical Sciences, Kumamoto University, Kumamoto, Japan; 32 Department of Gastroenterology and Hepatology, National Hospital Organization Fukuokahigashi Medical Center, Fukuoka, Japan; 33 Department of Medicine and Clinical Science, Graduate School of Medical Sciences, Kyushu University, Fukuoka, Japan; 34 Department of Endoscopy, Fukushima Medical University Hospital, Fukushima, Japan; 35 Division of Gastroenterology, Department of Internal Medicine, Iwate Medical University, Iwate, Japan; 36 Department of Internal Medicine, National Defense Medical College, Saitama, Japan; 37 Division of Endoscopy and Ultrasonography, Department of Clinical Pathology and Laboratory Medicine, Kawasaki Medical School, Kurashiki, Okayama Japan; 38 Department of Gastroenterology and Hepatology, Suita Municipal Hospital, Osaka, Japan; 39 Department of Endoscopy, University of the Ryukyus Hospital, Okinawa, Japan; 40 Department of Gastroenterology, National Hospital Organization Kyushu Medical Center, Fukuoka, Japan; 41 Department of Gastroenterological Endoscopy, Fukuoka University Hospital, Fukuoka, Japan; 42 Department of Gastroenterology, Kitasato University, School of Medicine, Kanagawa, Japan; 43 Department of Gastroenterology and Neurology, Akita University Graduate School of Medicine, Akita, Japan; 44 Digestive and Lifestyle Diseases, and Hygiene and Health Promotion Medicine, Kagoshima University Graduate School of Medical and Dental Sciences, Kagoshima, Japan; 45 Department of Gastroenterological Endoscopy, Tokyo Medical University, Tokyo, Japan; 46 Department of Gastroenterology and Hepatology, National Center for Global Health and Medicine, Tokyo, Japan

**Keywords:** Gastroenterology, Gastrointestinal diseases

## Abstract

The relationship between blood group and rebleeding in acute lower gastrointestinal bleeding (ALGIB) remains unclear. This study aimed to investigate the association between blood group O and clinical outcomes in patients with ALGIB. The study included 2336 patients with ALGIB whose bleeding source was identified during initial endoscopy (from the CODE BLUE-J Study). The assessed outcomes encompassed rebleeding and other clinical parameters. The rebleeding rates within 30 days in patients with blood group O and those without blood group O were 17.9% and 14.9%, respectively. Similarly, the rates within 1 year were 21.9% for patients with blood group O and 18.2% for those without blood group O. In a multivariate analysis using age, sex, vital signs at presentation, blood test findings, comorbidities, antithrombotic medication, active bleeding, and type of endoscopic treatment as covariates, patients with blood group O exhibited significantly higher risks for rebleeding within 30 days (odds ratio [OR] 1.31; 95% confidence interval [CI] 1.04–1.65; *P* = 0.024) and 1 year (OR 1.29; 95% CI 1.04–1.61; *P* = 0.020) compared to those without blood group O. However, the thrombosis and mortality rates did not differ significantly between blood group O and non-O patients. In patients with ALGIB, blood group O has been identified as an independent risk factor for both short- and long-term rebleeding.

## Introduction

Rebleeding is a serious problem associated with acute lower gastrointestinal bleeding (ALGIB). Previous reports have indicated that the incidence of rebleeding in patients with ALGIB within 30 days ranges from 5.2 to 26%^1–4^. Managing the risk of rebleeding is crucial in clinical practice, especially considering the current lack of preventive therapies for ALGIB, similar to the use of proton pump inhibitors for upper gastrointestinal bleeding (UGIB)^5^.

Blood group O has been associated with a high risk for various bleeding disorders^6^, including traumatic hemorrhage, UGIB, cerebral hemorrhage, postpartum hemorrhage, and epistaxis^7–11^. Additionally, individuals with blood group O are believed to have a low risk of thrombosis^12^. Recent findings also suggest that patients with blood group O may have an increased risk of bleeding following endoscopic procedures^13^. Although previous studies identified age, antithrombotic drug use, and blood transfusion as risk factors for ALGIB rebleeding^4,14^, the association between blood group and ALGIB rebleeding remains unknown.

To address these knowledge gaps, this multicenter study aimed to determine whether blood group O serves as an independent risk factor influencing the clinical outcomes of patients hospitalized for ALGIB. Furthermore, we explored the association between blood group and colonic diverticular bleeding (CDB), the most common cause of ALGIB^15^.

## Results

### Patient characteristics

**Figure 1:**
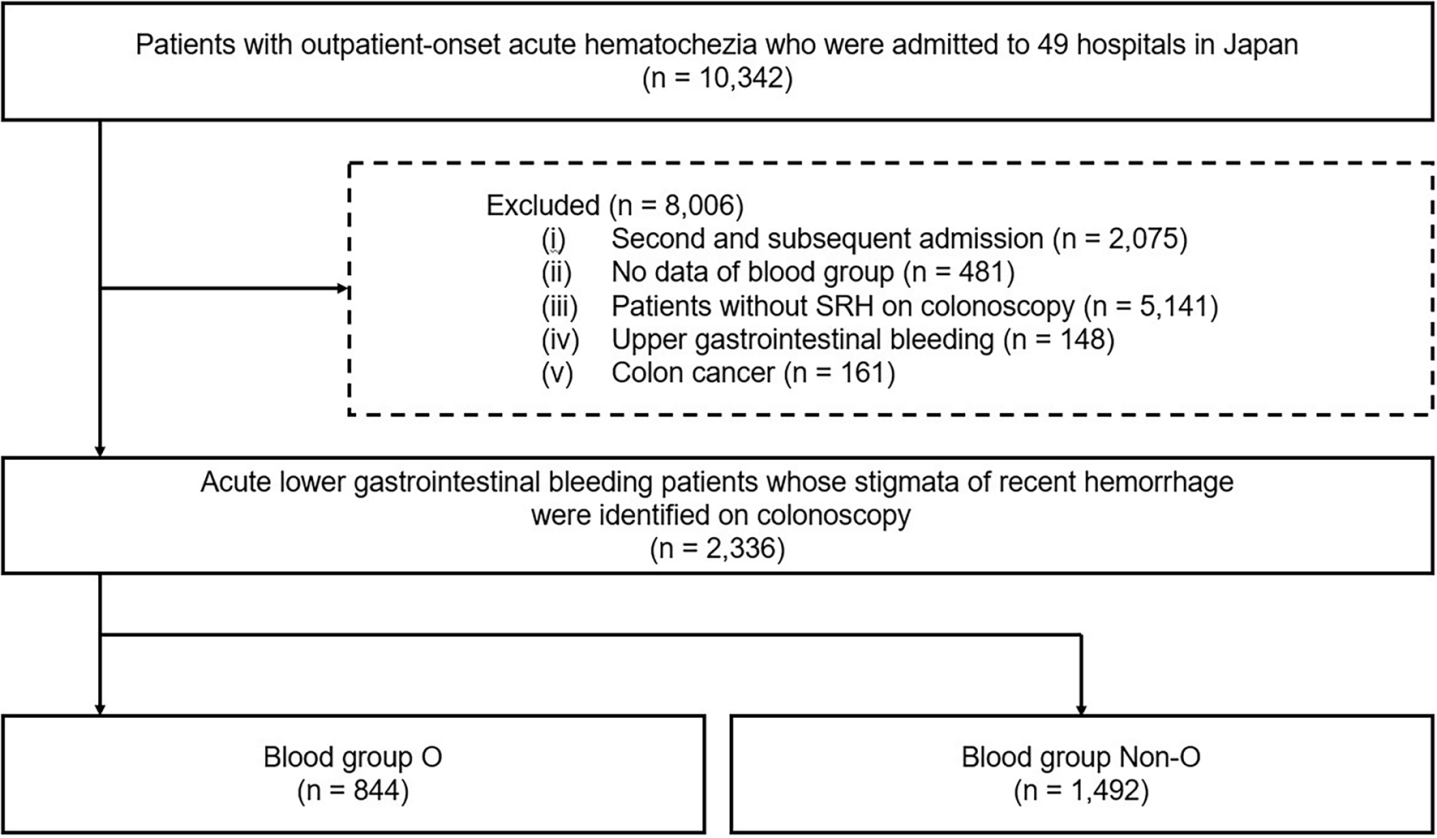
Study flow chart SRH, stigmata of recent hemorrhage.

A total of 10,342 patients admitted with ALGIB were included in the database. After excluding 8,006 patients based on the criteria outlined in Fig. 1, 2336 patients were eligible for analysis. These patients were categorized into two groups: 844 patients with blood group O and 1,492 patients with non-O blood groups (A, B, and AB). Among the ALGIB patients, 1,576 (67.5%) were male, and the mean age was 70.8 years ± 13.3 (mean ± standard deviation). The rebleeding rate in the ALGIB cohort was 22.5% (526/2,336 patients).

Table 1 displays the characteristics and clinical profiles of the enrolled patients stratified according to their ABO blood group status. When comparing the four groups (O, A, B, and AB), a significantly low proportion of patients with group O exhibited a modified Charlson Comorbidity Index (CCI) ≥ 2, platelet count < 150,000/μL, and blood urea nitrogen (BUN) > 25 mg/dL (*P* = 0.020, *P* = 0.005, *P* = 0.002, respectively). In comparison between the O and non-O (A, B, and AB) groups, a significantly low proportion of patients with group O were aged ≥ 65 years, had liver cirrhosis, heart failure, chronic kidney disease, and were using antithrombotic drugs. No significant differences were observed in other variables, including sex, body mass index (BMI), alcohol consumption, smoking, symptoms, and vital signs at admission, other laboratory data, or endoscopic factors.

**Table 1:** Baseline characteristics of acute lower gastrointestinal bleeding patients (*n* = 2,336).

Characteristics	Four-group comparison				*P* value	Two-group comparison		
	O (*n* = 844)	A (*n* = 876)	B(*n* = 419)	AB (*n* = 197)		O(*n* = 844)	non-O (*n* = 1,492)	*P* value
Age ≥ 65 years old	592 (70.1)	653 (74.5)	307 (73.2)	143 (72.6)	0.229	592 (70.1)	1103 (73.9)	**0.049**
Sex, male	564 (66.8)	598 (68.3)	278 (66.4)	136 (69.0)	0.832	564 (66.8)	1012 (67.8)	0.619
BMI ≥ 25	207 (26.3)	217 (26.3)	107 (26.9)	46 (25.0)	0.972	579 (73.7)	1038 (73.7)	0.977
Current drinker	364 (43.1)	374 (42.7)	167 (39.9)	81 (41.1)	0.265	364 (43.1)	622 (41.7)	0.324
Current smoker	144 (17.1)	138 (15.8)	61 (14.6)	29 (14.7)	0.511	144 (17.1)	228 (15.3)	0.200
Performance status ≥ 2	96 (11.5)	116 (13.4)	58 (14.0)	24 (12.4)	0.548	96 (11.5)	198 (13.4)	0.182
Comorbidities								
CCI ≥ 2	298 (35.3)	362 (41.3)	181 (43.2)	79 (40.1)	**0.020**	298 (35.3)	622 (41.7)	**0.002**
History of colorectal surgery	43 (5.1)	59 (6.7)	27 (6.4)	12 (6.1)	0.533	43(5.1)	98 (5.6)	0.151
History of colonic diverticular bleeding	211 (25)	212 (24.2)	93 (22.2)	57 (29.1)	0.312	211 (25.0)	362 (24.3)	0.704
Hypertension	480 (56.9)	495(56.5)	246 (58.7)	101 (51.3)	0.381	480 (56.9)	842 (56.4)	0.838
Diabetes mellitus	156 (18.5)	181 (20.7)	90 (21.5)	42 (21.3)	0.527	156 (18.5)	313 (21.0)	0.148
Dyslipidemia	208 (24.6)	219 (25.0)	109 (26.0)	53 (26.9)	0.890	208 (24.6)	381 (25.5)	0.627
Cirrhosis	6 (0.7)	17 (1.9)	11 (2.6)	4 (2.0)	0.051	6 (0.7)	32 (2.1)	**0.009**
Heart failure	65 (7.7)	93 (10.6)	47 (11.2)	17 (8.6)	0.108	65 (7.7)	157 (10.5)	**0.025**
Chronic kidney disease	107 (12.7)	135 (15.4)	72 (17.2)	30 (15.2)	0.158	107 (12.7)	237 (15.9)	**0.036**
Symptoms								
Loss of consciousness	63 (7.5)	63 (7.2)	30 (7.2)	14 (7.1)	0.995	63 (7.5)	107 (7.2)	0.794
Abdominal pain	50 (5.9)	54 (6.2)	31 (7.4)	18 (9.1)	0.340	50 (5.9)	103 (6.9)	0.355
Fever > 38	23 (2.7)	41 (4.7)	14 (3.3)	9 (4.6)	0.159	23 (2.7)	64 (4.3)	0.055
Medications								
NSAIDs	73 (8.7)	86 (9.8)	34 (8.1)	16 (8.1)	0.699	73 (8.7)	136 (9.1)	0.705
COX-2 selective inhibitors	17 (2.0)	16 (1.8)	15 (3.6)	5 (2.5)	0.228	17 (2.0)	36 (2.4)	0.534
Corticosteroids								
Antithrombotic drugs	335 (39.7)	385 (44.0)	190 (45.3)	86 (43.7)	0.175	335 (39.7)	661 (44.3)	**0.030**
Antiplatelet drugs	240 (28.4)	284 (32.4)	136 (32.5)	64 (32.5)	0.257	240 (28.4)	484 (32.4)	**0.044**
Anticoagulation drugs	136 (16.1)	154 (17.6)	79 (18.9)	31 (15.7)	0.596	136 (16.1)	264 (17.7)	0.330
Hemodynamics								
Systolic blood pressure < 100 mmHg	116 (13.7)	113 (12.9)	60 (14.3)	24 (12.2)	0.897	116 (13.7)	197 (13.2)	0.750
Heart rate > 100 bpm	177 (21.0)	163 (18.6)	87 (20.8)	32 (16.2)	0.420	177 (21.0)	282 (18.9)	0.244
Shock Index > 1	51 (6.0)	60 (6.8)	28 (6.7)	9 (4.6)	0.671	51 (6.0)	97 (6.5)	0.643
Laboratory data								
White blood cells > 10,000/μL	110 (13.1)	131 (15.0)	67 (16.0)	32 (16.2)	0.424	110 (13.1)	230 (15.4)	0.119
Hemoglobin < 12 g/dL	473 (56.0)	525 (59.9)	241 (57.5)	111 (56.3)	0.423	473 (56.0)	877 (58.8)	0.209
Platelet count < 15 × 10^4^/μL	118 (14.0)	178 (20.3)	79 (18.9)	32 (16.2)	**0.005**	118 (14.0)	289 (19.4)	**0.001**
Blood urea nitrogen > 25 mg/dL	164 (19.8)	227 (26.2)	118 (28.2)	47 (24.2)	**0.002**	164 (19.8)	392 (26.5)	**0.0003**
PT-INR > 1.5	72 (9.9)	96 (12.7)	40 (10.7)	17 (10.1)	0.370	72 (9.9)	153 (11.8)	0.205
Endoscopic treatment								
Clipping	455 (53.9)	470 (53.7)	236 (56.3)	100 (50.8)	0.618	455 (53.9)	806 (54.0)	0.959
Ligation	238 (28.2)	225 (25.7)	99 (23.6)	54 (27.4)	0.334	238 (28.2)	378 (25.3)	0.143

Data are presented as *n* (%). A two-tailed *P*-value < 0.05 was considered to indicate statistical significance. Bold values indicate *P* < 0.05. Analyzed using Fisher’s exact test.

Abbreviations: BMI, body mass index; CCI, Charlson comorbidity index; NSAIDs, Non-Steroidal Anti-Inflammatory Drugs; PT-INR, international normalized ratio of prothrombin time.

### Incidence of rebleeding according to ABO blood group

When comparing the four blood groups (O, A, B, and AB), the incidence rates of rebleeding during the observation period were 24.2%, 22.4%, 21.5%, and 18.3%, respectively (*P* = 0.305, χ2 test). The incidence rates of rebleeding within 30 days were 17.9% [151/844], 15.8% [138/876], 13.6% [57/419], and 14.2% [28/197], respectively (*P* = 0.209, χ2 test). Similarly, the incidence rates of rebleeding within 1 year were 21.9% [185/844], 19.3% [169/876], 17.0% [71/419], and 15.7% [31/197], respectively (*P* = 0.083, χ2 test). Although no statistically significant difference in the incidence rate of rebleeding was observed among the four groups, a tendency towards a high rebleeding rate was observed in individuals with blood group O.

### The rebleeding within 30 days and 1 year after the initial colonoscopy for ALGIB patients

**Figure 2:**
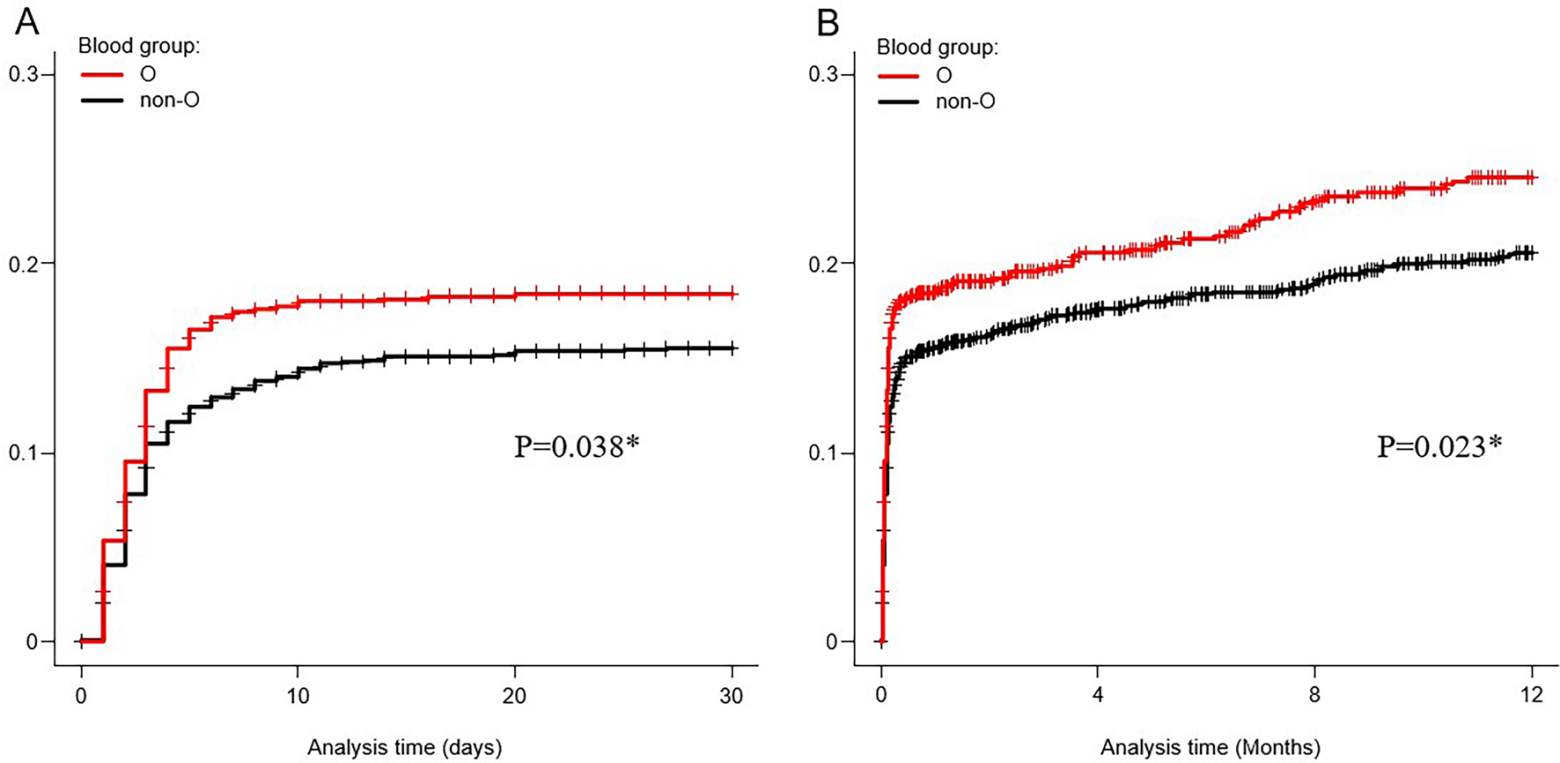
The cumulative probability of rebleeding using Kaplan–Meier method in acute lower gastrointestinal bleeding patients. (**A**) The cumulative probability of rebleeding within 30-day according to blood group (*n* = 2,336). (**B**) The cumulative probability of rebleeding within 1-year according to blood group (*n* = 2,336). * *P*-values were calculated using the Generalized Wilcoxon test.

Table 2 presents the rebleeding rates of patients with ALGIB within 30 days and 1 year after initial colonoscopy. In the univariate analysis, the risk of rebleeding within 30 days was higher in blood group O than in the non-O group (odds ratio [OR] 1.24 [95% confidence interval [CI] 0.99–1.55], *P* = 0.063), although not statistically significant. Blood group O was associated with an increased risk of rebleeding within 1 year (OR 1.26 [95% CI; 1.03–1.56], *P* = 0.028). In the multivariate analysis using a logistic regression model, after adjusting for other confounding factors, blood group O was confirmed as an independent risk factor for rebleeding within 30 days (adjusted OR [aOR] 1.31 [95% CI 1.04–1.65], *P* = 0.024) and within 1 year (aOR 1.29 [95% CI 1.04–1.61], *P* = 0.020). No significant associations were observed between the ABO blood group and rates of transfusion, active bleeding on colonoscopy, extravasation on computed tomography (CT), hospitalization for > 8 days, rate of interventional radiology (IVR), rate of surgery, incidence of thrombosis, or mortality within 30 days of colonoscopy. Kaplan–Meier analysis revealed a significantly higher cumulative probability of rebleeding within 30 days (*P* = 0.038, Generalized Wilcoxon test) and 1 year (*P* = 0.023, Generalized Wilcoxon test) in group O than in the non-O group among patients with ALGIB (Fig. 2). The median follow-up period for rebleeding was 413 days (interquartile range [IQR], 40–1109).

**Table 2:** Clinical outcomes of acute lower gastrointestinal bleeding patients (*n* = 2,336).

Clinical outcome	Group O (*n* = 844)	Group non-O (*n* = 1,492)	Crude OR (95% CI)	*P* value	Adjusted OR† (95% CI)	*P* value
Rebleeding within 30-day	151 (17.9)	223 (14.9)	1.24 (0.99–1.55)	0.063	1.31 (1.04–1.65)	**0.024**
Rebleeding within 1-year	185 (21.9)	271 (18.2)	1.26 (1.03–1.56)	**0.028**	1.29 (1.04–1.61)	**0.020**
Blood transfusion	277 (32.8)	493 (33.0)	0.99 (0.83–1.18)	0.912	1.14 (0.94–1.39)	0.168
Active bleeding	447 (53.0)	768 (51.5)	1.06 (0.90–1.26)	0.489	1.10 (0.92–1.31)	0.284
Extravasation on CT	186 (22.0)	337 (22.6)	0.97 (079–1.19)	0.760	0.92 (0.75–1.14)	0.455
Length of stay ≥ 8 days	428 (50.7)	755 (50.6)	1.00 (0.85–1.19)	0.960	1.09 (0.91–1.31)	0.333
Interventional radiology	11 (1.3)	26 (1.7)	0.75 (0.37–1.51)	0.416	0.77 (0.37–1.59)	0.479
Surgery	3 (0.4)	13 (0.9)	0.41 (0.12–1.43)	0.160	0.29 (0.06–1.30)	0.104
Thrombosis	6 (0.7)	9 (0.6)	1.18 (0.42–3.33)	0.755	1.49 (0.51–4.39)	0.470
Death within 30-day after colonoscopy	2 (0.2)	16 (1.1)	0.22 (0.05–0.96)	**0.043**	0.32 (0.07–1.42)	0.136

Values are the number and (%). A two-tailed *P*-value < 0.05 was considered to indicate statistical significance. Bold values indicate *P* < 0.05. Analyzed using Fisher’s exact test.

Each of the ORs is obtained by multivariate logistic regression analysis.

^†^ORs were adjusted for the contributing factors of clinically important variables: age ≥ 65, sex, Shock index > 1, active bleeding, ligation and the following 7 factors found to have significance (*P* < 0.1) on univariate analysis: fever > 38, heart failure, chronic kidney disease, cirrhosis, antithrombotic drugs, platelets < 15 × 10^4^/μL and blood urea nitrogen > 25 mg/dL.

CT, computed tomography; OR, odds ratio.

### The rebleeding within 30 days and 1 year after the initial colonoscopy for CDB patients

**Figure 3:**
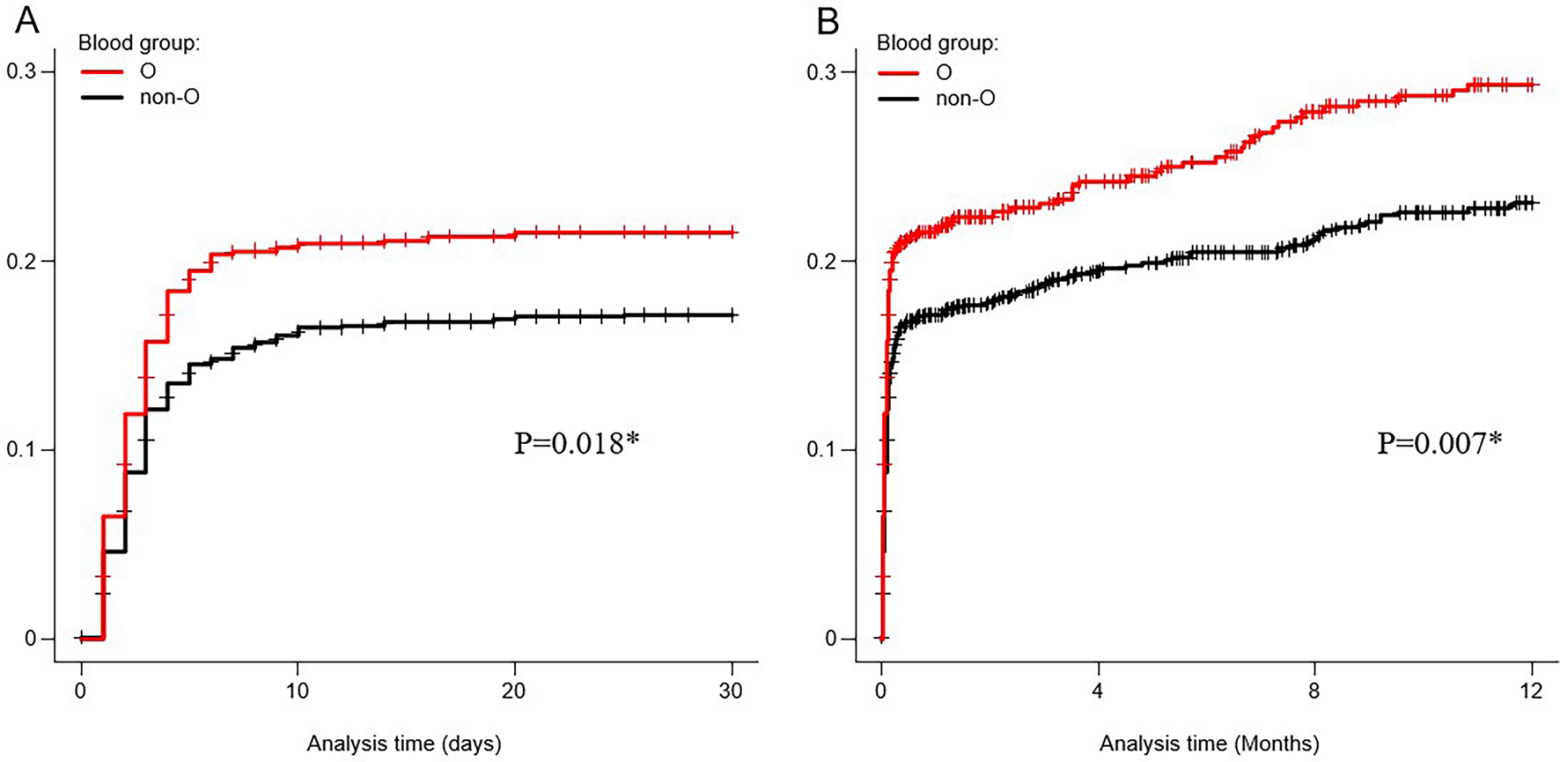
The cumulative probability of rebleeding using Kaplan–Meier method in colonic diverticular bleeding patients. (**A**) The cumulative probability of rebleeding within 30-day according to blood group (*n* = 1,650). (**B**) The cumulative probability of rebleeding within 1-year according to blood group (*n* = 1,650). * *P*-values were calculated using the Generalized Wilcoxon test.

Table 3 presents the rebleeding rates of patients with CDB within 30 days and 1 year after initial colonoscopy. In the cohort, approximately, 1,650 cases were diagnosed with CDB, which was the most frequent final diagnosis among patients with ALGIB (Table 4). In the univariate analysis, blood group O was identified as a risk factor for rebleeding within 30 days (OR 1.33 [95% CI 1.03–1.71], *P* = 0.029). Moreover, blood group o was also associated with an increased risk of rebleeding within 1 year (OR 1.36 [95% CI 1.08–1.72], *P* = 0.010). In the multivariate analysis using a logistic regression model, after adjusting for other confounding factors, blood group O was confirmed as an independent risk factor for rebleeding within 30 days (aOR 1.38 [95% CI 1.06–1.80], *P* = 0.016) and within 1 year (aOR 1.37 [95% CI 1.07–1.74], *P* = 0.012). Additionally, no significant association was observed between the ABO blood group and the rate of transfusion, active bleeding on colonoscopy, extravasation on CT, hospitalization for > 8 days, rate of IVR, rate of surgery, incidence of thrombosis, or mortality within 30 days of colonoscopy. Kaplan–Meier analysis revealed a significantly higher cumulative probability of rebleeding within 30 days (*P* = 0.018, Generalized Wilcoxon test) and 1 year (*P* = 0.007, Generalized Wilcoxon test) in the group with blood group O compared to the non-O group among patients with CDB (Fig. 3).

**Table 3:** Clinical outcomes of colonic diverticular bleeding patients (*n* = 1,650).

Clinical outcome	Group O (*n* = 614)	Group non-O (*n* = 1,036)	Crude OR (95% CI)	*P* value	Adjusted OR† (95% CI)	*P* value
Rebleeding within 30-day	129 (21.0)	173 (16.7)	1.33 (1.03–1.71)	**0.029**	1.38 (1.06–1.80)	**0.016**
Rebleeding within 1-year	160 (26.1)	213 (20.6)	1.36 (1.08–1.72)	**0.010**	1.37 (1.07–1.74)	**0.012**
Blood transfusion	216 (35.2)	348 (33.6)	1.07 (0.87–1.32)	0.511	1.22 (0.97–1.53)	0.084
Active bleeding	335 (54.6)	548 (52.9)	1.07 (0.88–1.31)	0.512	1.11 (0.91–1.37)	0.307
Extravasation on CT	168 (27.4)	292 (28.2)	0.96 (0.77–1.20)	0.718	0.92 (0.73–1.16)	0.475
Length of stay ≥ 8 days	340 (55.4)	567 (55.7)	1.03 (0.84–1.25)	0.799	1.10 (0.89–1.37)	0.366
Interventional radiology	11 (1.8)	25 (2.4)	0.74 (0.36–1.51)	0.405	0.80 (0.38–1.66)	0.548
Surgery	1 (0.2)	4 (0.4)	0.42 (0.05–3.77)	0.439	0.36 (0.04–3.31)	0.364
Thrombosis	5 (0.8)	7 (0.7)	1.21 (0.38–3.82)	0.749	1.59 (0.47–5.42)	0.456
Death within 30-day after colonoscopy	2 (0.3)	3 (0.3)	1.13 (0.19–6.75)	0.897	1.33 (0.21–8.41)	0.760

Values are the number and (%). A two-tailed *P*-value < 0.05 was considered to indicate statistical significance. Bold values indicate *P* < 0.05. Analyzed using Fisher’s exact test.

Each of the ORs is obtained by multivariate logistic regression analysis.

^†^ORs were adjusted for the contributing factors of clinically important variables: age ≥ 65, sex, Shock index > 1, active bleeding, ligation, and the following 7 factors found to have significance (*P* < 0.1) on univariate analysis: fever > 38, heart failure, chronic kidney disease, cirrhosis, antithrombotic drugs, platelets < 15 × 10^4^/μL and blood urea nitrogen > 25 mg/dL.

CT, computed tomography; OR, odds ratio.

**Table 4:** Final diagnosis of acute lower gastrointestinal bleeding with stigmata of recent hemorrhage (*n* = 2,336).

Diagnosis	Total (*n* = 2,336)	Four-group comparison				*P* value	Two-group comparison		*P* value
		O (*n* = 844)	A (*n* = 876)	B (*n* = 419)	AB (*n* = 197)		O (*n* = 844)	non-O (*n* = 1,492)	
Colonic diverticular bleeding	1,650 (71)	614 (72.8)	612 (69.9)	288 (68.7)	136 (69.0)	0.385	614 (72.8)	1,036 (69.4)	0.091
Postprocedure bleeding	279 (12)	105 (12.4)	106 (12.1)	42 (10.0)	26 (13.2)	0.576	105 (12.4)	174 (11.7)	0.577
Rectal ulcer	123 (5)	34 (4.0)	52 (5.9)	25 (6.0))	12 (6.1)	0.255	34 (4.0)	89 (6.0)	**0.044**
Colorectal angioectasia	52 (2)	14 (1.7)	19 (2.2)	17 (4.1)	2 (1.0)	**0.029**	14 (1.7)	38 (2.6)	0.162
Radiation colitis	28 (1)	13 (1.5)	8 (0.9)	5 (1.2)	2 (1.0)	0.685	13 (1.5)	15 (1.0)	0.254
Others	204 (8.7)	64 (7.6)	79 (9.0)	42 (10.0)	19 (9.6)	0.463	64 (7.6)	140 (9.4)	0.138

Data are presented as *n* (%). A two-tailed *P*-value < 0.05 was considered to indicate statistical significance.Bold values indicate *P* < 0.05. Analyzed using Fisher’s exact test.

Others includes the following diseases; small intestinal bleeding including presumptive cases, ischemic colitis, hemorrhoid bleeding, infectious colitis, inflammatory bowel disease, Meckel's diverticulum bleeding, drug-induced colonic ulcer.

## Discussion

The findings from this nationwide cohort study provide novel evidence that the risk of rebleeding in patients with ALGIB and CDB varies according to the blood group (Tables 2, 3). Notably, supplementary analysis revealed higher short- and long-term cumulative rebleeding rates in patients with ALGIB and CDB in O group than in those in the non-O group (Figs. 1, 2). Conversely, for clinical outcomes other than rebleeding, such as the incidence of thrombosis and short-term mortality, the association with blood group O was not statistically significant (Tables 2, 3). In contrast, analysis of similar clinical outcomes in patients with non-diverticular bleeding showed no significant differences based on blood group (Supplementary Table 1). Overall, the results of this retrospective multicenter study indicate that blood grouping is pertinent not only for transfusion compatibility but also for assessing the risk of rebleeding in ALGIB, particularly in CDB.

In this study, individuals with blood group O experienced a higher incidence of rebleeding than those with the non-O group despite exhibiting few risk factors for rebleeding, such as age, platelet count, and use of antithrombotic drugs. This suggests that individuals with blood group O may have additional risk factors for rebleeding. Previous studies have elucidated the underlying mechanism of the association between blood group O and bleeding, indicating a von Willebrand factor (VWF) deficiency. Serum levels of VWF in individuals with blood group O are 25–35% lower than those in individuals with non-O blood group^16–20^. VWF plays a crucial role in primary hemostasis by bridging injured vascular endothelium and platelets and in secondary hemostasis by activating fibrin clot formation as a factor VIII transport protein. In individuals with a non-O blood group, VWF glycosylation protects them from proteolysis and/or clearance by ADAMTS13 (a disintegrin and metalloproteinase with a thrombospondin type 1 motif, member 13) and LRP1 (low-density lipoprotein receptor-related protein 1), respectively, resulting in elevated VWF levels compared to those in individuals with blood group O^21,22^. Furthermore, coagulation factor VIII activity is reported to be low in blood group O^23,24^. Additionally, platelet function, as analyzed using PFA-100^25^, is also reduced in individuals with blood group O. These findings provide a theoretical explanation for the diminished hemostatic capacity observed in individuals with blood group O.

Supporting this hypothesis, previous clinical studies have demonstrated that blood group O is a notable risk factor linked to gastrointestinal bleeding^7,8,10,26,27^. Furthermore, in a meta-analysis, blood group O was identified as an independent risk factor (OR, 1.33) for various bleeding disorders including skin bleeding, cerebral hemorrhage, postpartum hemorrhage, and epistaxis^9^. Conversely, documented evidence exists that thrombotic events (e.g. deep venous thromboembolism and coronary heart disease) are less prevalent in individuals with blood group O^28–31^. This reveals discernible differences in clinical outcomes between individuals with blood group O and those without. Genome-wide association analyses have reported a correlation between ABO genes and colonic diverticular disease^32–34^, suggesting a potential association between blood groups and CDB.

In this study, the discrepancy in cumulative rebleeding rates between patients with blood group O and those with non-O blood groups was most pronounced during the initial week post-admission, after which it maintained a relatively stable trajectory. This observed pattern aligns with the findings of a study that examined the relationship between blood group O and delayed bleeding following endoscopic procedures^13^, implying the potential influence of analogous mechanisms. The intrinsic primary hemostatic function in individuals with blood group O may be comparatively delicate, potentially explaining the observed surge in early rebleeding events. Once effective thrombogenesis is established, its effect on the fibrinolytic system appears minimal and does not contribute to an increase in long-term rebleeding rates. This phenomenon likely underlies the observed stabilization of the cumulative rebleeding rates over time. The management of rebleeding is crucial in the early stages of ALGIB.

This study has inherent limitations that should be considered. First, this was a retrospective multicenter cohort study utilizing a large dataset compiled from medical records spanning the past 10 years across 49 hospitals, including large academic facilities throughout Japan. However, a detailed investigation of the bleeding site was not performed. Second, while the use of antithrombotic drugs may pose a risk of rebleeding in patients with ALGIB, this study was only able to collect information on the use of antithrombotic drugs at the time of admission. To address this issue, we conducted a sensitivity analysis on the rebleeding risk of blood group O, focusing on cases in which antithrombotic drug status and management (no use/withdrawal/resumption/continuation) were confirmed (*n* = 1829), excluding patients missing information on antithrombotic drug management (*n* = 507). The main results were unchanged after adjusting for antithrombotic drug management, confirming their robustness (Supplementary Table 2). Third, although our findings confirmed that blood group O is a risk factor for rebleeding in ALGIB, whether the particular blood group is also a risk factor for initial bleeding events remains unclear.

In conclusion, this study demonstrated that blood group O is an independent risk factor for both short- and long-term rebleeding in patients with ALGIB. Notably, the findings from this retrospective multicenter analysis suggest that blood grouping is significant not only for transfusion compatibility but also for its implications in assessing the risk of rebleeding in ALGIB. Therefore, rebleeding should be managed with heightened caution in patients with blood group O who present with ALGIB.

## Methods

### Patients and study design

This study included 10,342 patients admitted to 49 hospitals for acute hematochezia between January 2010 and December 2019. From the original cohort of patients with acute hematochezia (*n* = 10,342), we excluded those with second and subsequent admissions (*n* = 2075), those lacking blood group data (*n* = 481), those without a definitive diagnosis on colonoscopy based on stigmata of recent hemorrhage (SRH) (*n* = 5,141), those with UGIB (*n* = 148), and those with colon cancer (*n* = 161). The remaining cohort was stratified into two groups: blood group O and non-O. The present study was conducted in accordance with the Declaration of Helsinki of 1964 and its subsequent amendments, or equivalent ethical standards. As this study is a retrospective investigation, the requirement for obtaining informed consent from patients was waived by the central institution, Tokyo Medical University. The central institution has a license committee/institutional review board (IRB) to approve research involving human subjects. The research protocol obtained approval from the Tokyo Medical University IRB (T20190244). The central institution's IRB review was applied to this study, and approval was obtained from the ethics committees and IRB of all participating hospitals (Supplementary Table 3).

### Variables

All variables were extracted from medical records and electronic endoscopy databases of each participating institution by gastroenterologists or researchers. A total of 42 items, including baseline characteristics such as blood group, age, sex, BMI, drinking habits, smoking, performance status, comorbidities, clinical symptoms, medications, vital signs on admission, initial laboratory findings, endoscopy, and CT results, were collected as previously documented^15,35^. Comorbidities were evaluated using the modified CCI^36^, which includes 19 conventional CCI items and additional items for hypertension and hyperlipidemia. The CCI serves as an index for categorizing comorbidities with prognostic relevance and has been extensively validated for gastrointestinal bleeding^37^. The shock index was defined as heart rate divided by systolic blood pressure^38^. Contrast-enhanced CT was used to assess extravasation. Detailed information on the endoscopic factors, including the type of SRH and endoscopic treatment, was also collected.

### Clinical outcomes

The primary outcome was rebleeding within 30 days and 1 year after the initial colonoscopy. Rebleeding was defined as the presence of a large volume of fresh blood or dark-red stool after the initial colonoscopy^15,39^. Secondary outcomes included various clinical features, such as blood transfusion, active bleeding, extravasation on CT, length of stay, IVR, surgery, thrombosis, and death within 30 days post-colonoscopy.

### Statistical analysis

The association between clinical variables and blood groups was assessed in patients with ALGIB. The analysis included four groups: O, A, B, and AB, and a two-group analysis comparing the O and non-O groups. Categorical data were compared using the χ2 test or Fisher’s exact test. The relationship between blood group and clinical outcomes was analyzed using univariate and multivariate logistic regression models in the two-group comparison (O vs. non-O). The multivariate analysis was adjusted to include clinically significant variables (age ≥ 65, gender, Shock index > 1, active bleeding, and endoscopic ligation) and variables with at least borderline significance (*P* < 0.10) in univariate analysis. The cumulative incidence of rebleeding was analyzed using the generalized Wilcoxon test with the Kaplan–Meier method. In the subgroup analysis, we restricted the analysis to patients with CDB and performed similar examinations. This subgroup analysis aimed to elucidate the relationship between the blood group and the risk of rebleeding in CDB, a major cause of ALGIB. Statistical significance was defined as a two-sided *P*-value of < 0.05. Statistical analysis was conducted using JMP® Pro 16.0.0 (SAS Institute, Cary, NC, USA).

### Supplementary Information

Supplementary Tables.

## Data Availability

The datasets generated and/or analyzed during the current study are not publicly available due to privacy and ethical restrictions but can be requested and reviewed from the corresponding author on reasonable request.
