# Supplementary material for: Association of blood group O with a recurrent risk for acute lower gastrointestinal bleeding from a multicenter cohort study
Source: Sci Rep. 2024 Jun 17;14:13983. doi: 10.1038/s41598-024-64476-9 (PMC11183064; doi:10.1038/s41598-024-64476-9)
Supplement: Supplementary file 1 — Supplementary Tables. [file 41598_2024_64476_MOESM1_ESM.docx]

**Supplementary Table 1. Clinical outcomes of non-diverticular bleeding patients (n = 686)**

| Clinical outcome | Group O  (n = 230) | Group non-O  (n = 456) | Crude OR  (95% CI) | P value | Adjusted OR†  (95% CI) | P value |
| --- | --- | --- | --- | --- | --- | --- |
| Rebleeding within 30-day | 22 (9.6) | 50 (11.0) | 0.86 (0.51-1.46) | 0.573 | 1.01 (0.58-1.75) | 0.976 |
| Rebleeding within 1-year | 25 (10.9) | 58 (12.7) | 0.84 (0.51-1.38) | 0.483 | 0.98 (0.58-1.65) | 0.941 |
| Blood transfusion | 61 (26.5) | 145 (31.8) | 0.77 (0.54-1.10) | 0.155 | 0.91 (0.61-1.36) | 0.639 |
| Active bleeding | 118 (51.3) | 236 (51.8) | 1.02 (0.74-1.40) | 0.911 | 1.06 (0.75-1.48) | 0.743 |
| Extravasation on CT | 18 (7.8) | 45 (9.9) | 0.78 (0.44-1.37) | 0.383 | 0.80 (0.43-1.45) | 0.457 |
| Length of stay ≥ 8 days | 88 (38.3) | 188 (41.2) | 0.88 (0.64-1.22) | 0.455 | 1.02 (0.71-1.45) | 0.924 |
| Interventional radiology | 0 (0) | 1 (0.2) | NA | NA | NA | NA |
| Surgery | 2 (0.9) | 9 (2) | 0.44 (0.09-2.03) | 0.291 | 0.22 (0.03-1.79) | 0.156 |
| Thrombosis | 1 (0.4) | 2 (0.4) | 0.99 (0.09-10.99) | 0.994 | 1.61 (0.09-28.36) | 0.745 |
| Post colonoscopy death within 30-day | 0 (0) | 13 (2.9) | NA | NA | NA | NA |

Note: Values are the number and (%). A two-tailed P-value<0.05 was considered to indicate statistical significance. Each of the ORs is obtained by multivariate logistic regression analysis.

†ORs were adjusted for the contributing factors of clinically important variables: age ≥65, sex, Shock index >1, active bleeding, EBL or snare use for endoscopic treatment and the following 7 factors found to have significance (P < 0.1) on univariate analysis: fever >38, heart failure, chronic kidney disease, cirrhosis, antithrombotic drugs, platelets <15 x 10^4^/μL and blood urea nitrogen >25 mg/dL.

Abbreviations: CT, computed tomography; OR, odds ratio; NA, not applicable

**Supplementary Table 2.** **Rebleeding risk of blood group O within 30-day and 1-year for acute lower gastrointestinal bleeding, colonic diverticular bleeding and non-diverticular bleeding patients after excluding patients missing information on antithrombotic drug management (Sensitivity analysis).**

|  | Clinical outcome | Group O | Group non-O | Crude OR  (95% CI) | P value | Adjusted OR†  (95% CI) | P value |
| --- | --- | --- | --- | --- | --- | --- | --- |
| ALGIB  (n = 1829) | Rebleeding within 30-day | 118/681 (17.3) | 165/1148 (14.4) | 1.25 (0.96-1.62) | 0.092 | 1.30 (0.99-1.69) | 0.056 |
|  | Rebleeding within 1-year | 149/681 (21.9) | 200/1148 (17.4) | 1.33 (1.05-1.68) | **0.019** | 1.34 (1.05-1.72) | **0.018** |
| Colonic diverticular bleeding  (n = 1283) | Rebleeding within 30-day | 103/490 (21.0) | 132/793 (16.6) | 1.33 (1.001-1.78) | **0.0495** | 1.38 (1.02-1.86) | **0.035** |
|  | Rebleeding within 1-year | 132/490 (26.9) | 161/793 (20.3) | 1.45 (1.11-1.88) | **0.006** | 1.46 (1.11-1.92) | **0.007** |
| Non-diverticular bleeding  (n = 546) | Rebleeding within 30-day | 15/191 (7.9) | 33/355 (9.3) | 0.83 (0.44-1.57) | 0.571 | 0.95 (0.49-1.87) | 0.891 |
|  | Rebleeding within 1-year | 17/191 (8.9) | 39/355 (11.0) | 0.79 (0.43-1.44) | 0.445 | 0.88 (0.46-1.65) | 0.680 |

Note: Values are the number and (%). A two-tailed P-value<0.05 was considered to indicate statistical significance. Each of the ORs is obtained by multivariate logistic regression analysis.

†ORs were adjusted for the contributing factors of clinically important variables: age ≥65, sex, Shock index >1, active bleeding, EBL or snare use for endoscopic treatment and the following 7 factors found to have significance (P < 0.1) on univariate analysis: fever >38, heart failure, chronic kidney disease, cirrhosis, antithrombotic drugs (no use/ withdrawal/ resumption/ continuation), platelets <15 x 10^4^/μL and blood urea nitrogen >25 mg/dL.

Categorical data of antithrombotic drug information for ALGIB patients is below: no use (n = 1379), withdrawal (n = 60), resumption (n = 225), continuation (n = 165).

Abbreviations: ALGIB, acute lower gastrointestinal bleeding; OR, odds ratio

**Supplementary Table 3. Affiliations, ethics committee approval number, and number of cases at the 49 participating hospitals in the CODE BLUE J-study**

| **No.** | **Prefecture** | **Institutions** | **Department** | **Ethics committee approval number** |
| --- | --- | --- | --- | --- |
| 1 | Tokyo | Tokyo Medical University | Department of Gastroenterological Endoscopy | T20190244 |
| 2 | Tokyo | National Center for Global Health and Medicine | Department of Gastroenterology and Hepatology | 3539 |
| 3 | Tokyo | Tokyo Shinagawa Hospital | Department of Gastroenterology | 20-A-04 |
| 4 | Tokyo | Nippon Medical School, Graduate School of Medicine | Department of Gastroenterology | B-2020-147 |
| 5 | Chiba | Chiba Hokusoh Hospital, Nippon Medical School | Department of Gastroenterology | 802 |
| 6 | Saga | Saga-Ken Medical Centre Koseikan | Department of Gastroenterology | 20-01-01-03 |
| 7 | Tokyo | St. Luke’s International University | Department of Gastroenterology | 20-R012 |
| 8 | Okayama | Kawasaki Medical School | Division of Endoscopy and Ultrasonography, Department of Clinical Pathology and Laboratory Medicine | 3890 |
| 9 | Okayama | Kawasaki Medical School General Medical Center | Division of Endoscopy and Ultrasonography, Department of Clinical Pathology and Laboratory Medicine | 3890 |
| 10 | Ibaraki | University of Tsukuba | Department of Gastroenterology and Division of Endoscopic Center | R02-030 |
| 11 | Tokyo | Tokyo Metropolitan Bokutoh Hospital | Department of Gastroenterology | 02-024 |
| 12 | Kanagawa | Saiseikai Yokohamashi Tobu Hospital | Emergency and Critical Care Center | 20200030 |
| 13 | Tokyo | The University of Tokyo | Department of Gastroenterology | 2020067NI |
| 14 | Tokyo | Toranomon Hospital | Department of Gastroenterology | 2021 |
| 15 | Aichi | Nagoya University Hospital | Department of Endoscopy | 2020-0152 |
| 16 | Hiroshima | Hiroshima City Asa Citizens Hospital | Department of Gastroenterology | 02-1-24 |
| 17 | Fukuoka | National Hospital Organization Fukuokahigashi Medical Center | Department of Gastroenterology and Hepatology | 2020-臨-2 |
| 18 | Nara | Nara City Hospital | Department of Gastroenterology and Hepatology and Center for Digestive and Liver Diseases | NCH倫20-8 |
| 19 | Niigata | Graduate School of Medical and Dental Sciences, Niigata University | Division of Gastroenterology | 2020-0052 |
| 20 | Kanagawa | St. Marianna University School of Medicine | Division of Gastroenterology and Hepatology, Department of Internal Medicine | 4802 |
| 21 | Oita | Oita University | Department of Gastroenterology | 1845 |
| 22 | Tokyo | Tokyo Saiseikai Central Hospital | Department of Internal Medicine | 2020-015-01 |
| 23 | Fukuoka | Fukuoka University Hospital | Department of Gastroenterological Endoscopy | U20-05-016 |
| 24 | Fukuoka | Fukuoka University Chikushi Hospital | Department of Gastroenterology | C20-052 |
| 25 | Osaka | Kitano Hospital, Tazuke Kofukai Medical Research Institute | Department of Gastroenterology and Hepatology | P200500400 |
| 26 | Fukuoka | Graduate School of Medical Sciences, Kyushu University | Department of Medicine and Clinical Science | 2020-289 |
| 27 | Miyazaki | University of Miyazaki Hospital | Department of Gastroenterology and Hepatology, Center for Digestive Disease, and Division of Endoscopy | 0-0734 |
| 28 | Okinawa | University of the Ryukyus Hospital | Department of Endoscopy | 1656 |
| 29 | Okinawa | Naha City Hospital | Department of Gastroenterology | 2004a4 |
| 30 | Kagoshima | Kagoshima University Graduate School of Medical and Dental Sciences | Digestive and Lifestyle Diseases | 200041疫 |
| 31 | Kagoshima | Kagoshima City Hospital | Department of Gastroenterology | 2020-25 |
| 32 | Kagoshima | Kagoshima Kouseiren Hospital | Department of Gastroenterology | 215 |
| 33 | Kagoshima | Kagoshima Medical Center | Department of Gastroenterology | 2020-22 |
| 34 | Kagoshima | Izumi General Medical Center | Department of Gastroenterology | 60 |
| 35 | Kagoshima | Kirishima City Medical Association Medical Center | Department of Gastroenterology | 202005 |
| 36 | Kagoshima | Kagoshima Prefectural Oshima Hospital | Department of Gastroenterology | 97 |
| 37 | Kyoto | National Hospital Organization Kyoto Medical Center | Department of Gastroenterology | 20-020 |
| 38 | Fukushima | Fukushima Medical University | Department of Gastroenterology | 一般2020-112 |
| 39 | Tokyo | Tokyo Metropolitan Cancer and Infectious Diseases Center Komagome Hospital | Department of Gastroenterology | 2503 |
| 40 | Kanagawa | Kitasato University, School of Medicine | Department of Gastroenterology | C20-174 |
| 41 | Osaka | Suita Municipal Hospital | Department of Gastroenterology and Hepatology | 2020-研2 |
| 42 | Akita | Akita University Graduate School of Medicine | Department of Gastroenterology and Neurology | 2491 |
| 43 | Shizuoka | Japanese Red Cross Shizuoka Hospital | Department of Gastroenterology | 2020-06 |
| 44 | Aomori | Hirosaki University Hospital | Division of Endoscopy | 2020-32 |
| 45 | Kumamoto | Graduate School of Medical Sciences, Kumamoto University | Department of Gastroenterology and Hepatology | 2040 |
| 46 | Fukuoka | National Hospital Organization Kyushu Medical Center | Department of Gastroenterology | 20C065 |
| 47 | Iwate | Iwate Medical University | Department of Internal Medicine | MH2020-050 |
| 48 | Yamaguchi | Shuto General Hospital | Department of Gastroenterology | H31-24 |
| 49 | Saitama | National Defense Medical College | Department of Internal Medicine | 4217 |
